# Consumer and Patient Health Information Seeking With Generative AI Tools: Scoping Review of Facilitators and Barriers

**DOI:** 10.2196/93944

**Published:** 2026-09-02

**Authors:** Lilach Alon, Inbar Levkovich

**Affiliations:** 1 Tel Hai Academic College Kiryat Shmona, Northern District Israel

**Keywords:** adoption, consumer health informatics, credibility, generative AI, health information seeking, large language model, LLM, Preferred Reporting Items for Systematic Reviews and Meta-Analyses Extension for Scoping Reviews, PRISMA-ScR, scoping review, trust, verification

## Abstract

**Background:**

Generative AI (GenAI) tools powered by large language models (LLMs) are increasingly used by the public to seek health information. Unlike traditional web search, these systems generate conversational responses that may alter how users assess credibility, manage uncertainty, verify information, and decide whether to consult clinicians. As GenAI becomes more embedded in everyday health information practices, a clearer synthesis of the emerging empirical evidence is needed.

**Objective:**

This scoping review mapped and synthesized empirical research on consumer and patient health information seeking using GenAI and LLM tools, with a focus on study contexts, outcome constructs, and the facilitators and barriers shaping use, reliance, and verification.

**Methods:**

The review adhered to Joanna Briggs Institute guidance for scoping reviews and reported using PRISMA-ScR (Preferred Reporting Items for Systematic Reviews and Meta-Analyses extension for Scoping Reviews), with search reporting additionally guided by PRISMA-S (Preferred Reporting Items for Systematic Reviews and Meta-Analyses Search Extension). We searched PubMed, Scopus, PsycINFO, Web of Science, IEEE Xplore, ACM Digital Library, Google Scholar, ERIC, EBSCO, and ProQuest for English-language studies published 2022 onward. The final updated search was conducted on January 8, 2026. Eligible studies were empirical quantitative, qualitative, or mixed methods studies examining health information seeking mediated by GenAI and LLM systems, wherein an LLM served as the interface or source for obtaining health information. Data were charted using a structured extraction form capturing study characteristics, populations, health contexts, GenAI tool types, outcomes, and factors shaping use.

**Results:**

The review included 27 studies. GenAI was used for symptom appraisal, condition understanding, treatment options, and care navigation. Reported facilitators included convenience and clarity, particularly efficiency and access (n=8, 29.6%), comprehensibility and presentation quality (n=11, 40.7%), personalization and specificity (n=5, 18.5%), and affective or interpersonal comfort (n=5, 18.5%). Reported barriers were dominated by credibility and trust concerns (n=13, 48.1%), particularly when accuracy cues or citations were missing or difficult to interpret. Additional barriers included perceived unsuitability for complex, urgent, or emotionally charged situations (n=5, 18.5%); privacy or data security concerns (n=4, 14.8%); limited prompting skills (n=2, 7.4%); and modality or interaction constraints that hindered credibility assessment and information comparison (n=5, 18.5%). Six (22.2%) studies reported literacy-related capability was, and 5 (18.5%) reported verification-supporting features, such as visible sourcing, transcripts, and save, revisit, or share functions.

**Conclusions:**

This review is innovative in focusing on health information seeking as a user practice rather than on technical performance or clinical implementation alone. Unlike prior reviews, it maps how the emerging literature conceptualizes use, trust, reliance, and verification. It contributes a structured synthesis of the main facilitators, barriers, and verification-related features reported on GenAI-mediated health information seeking. In practice, the findings suggest that safer use may depend on not only model quality but also users’ ability to interpret, verify, and act on AI-generated responses.

## Introduction

### Rationale

Generative AI (GenAI) systems powered by large language models (LLMs) are reshaping the health landscape by accelerating how medical knowledge is produced, summarized, and made accessible through conversational interfaces [1,2]. As these systems are embedded across consumer-facing platforms and health contexts, they increasingly function as intermediaries between lay users and complex clinical information. These systems offer synthesized explanations, definitions of terminology, and guidance for formulating follow-up questions in ways that can make health-related inquiries feel faster and more tractable than navigating multiple documents [3,4]. When people use GenAI for health questions, they often receive a single coherent response in a conversational format rather than a set of sources to compare, which can shift how credibility is judged and how uncertainty is managed within the interaction [5-7].

These shifts have immediate implications for clinician-patient interaction. As GenAI becomes part of everyday health information seeking, it can shape what patients believe is relevant, what they expect from clinical encounters, and how they negotiate next steps, even when the evidentiary basis of an AI-generated explanation is uncertain [8]. The stakes of trust calibration are therefore heightened because GenAI-mediated interpretations may enter consultations and inform self-care decisions, requests for tests, and perceptions of urgency [9,10]. In this setting, the clinical impact of GenAI depends not only on what information is produced but also on whether the interaction supports appropriate verification, communicates uncertainty, and keeps provenance inspectable enough to enable constructive dialogue with clinicians [4].

At the same time, GenAI is altering how people obtain health-related information outside the clinic. Conventional web searching requires users to navigate multiple sources and integrate sometimes conflicting claims. By contrast, LLM interfaces foreground a single synthesized answer and encourage iterative follow-up, which can compress evaluation and shift attention toward internal cues such as clarity, coherence, specificity, and apparent confidence [11,12]. Provenance and triangulation can become less visible unless the system makes sourcing easy to inspect and supports revisiting or comparing information across turns [13]. As a result, GenAI can change which information is accessed as well as the practical work required to evaluate it within the flow of seeking.

Understanding why these developments matter requires situating GenAI use within health information-seeking behavior. Health information seeking is commonly defined as purposive efforts to obtain and use health-related information to interpret symptoms, understand conditions and options, and guide decisions about self-care and professional consultation [14]. In practice, seeking is often triggered by decision-relevant uncertainty [15,16], such as whether a symptom warrants medical attention, how urgent the situation might be, or what the next step is [17,18]. Seeking may also be initiated by curiosity aimed at locating relevant information, clarifying concepts, or building an initial mental model of a condition [19].

These processes frequently unfold under constraints that can narrow deliberation and increase reliance on heuristic strategies. Health anxiety, fatigue, and information overload can reduce the capacity for careful evaluation and increase the preference for low-effort channels [2,20]. Individual resources such as eHealth literacy also vary substantially, shaping how people adopt and use AI-mediated health information and how effectively they assess or verify what they receive [21-23].

When evaluation is difficult, users often shift away from sustained source comparison and toward simpler compensatory strategies. These can include rephrasing queries, checking an additional channel, or switching to screen-based search to regain inspectable cues and external markers of credibility [13,16]. Trust is also shaped by whether the interface supports evaluation through affordances such as rereading, comparison, sharing, saving, and revisiting, as well as by mechanisms that make sourcing and uncertainty easier to interpret during use [6,12]. In an LLM interface that produces a single coherent synthesis, the temptation to treat the response as sufficient for orientation can further compress evaluation, particularly when users experience the interaction as efficient and emotionally easier to engage with [4,5,7]. Even when users anticipate possible errors, AI-generated information may still be carried into decisions and consultations, reinforcing the importance of systems that make corroboration feasible within the interaction rather than optional in principle [4,21].

Against this background, a growing body of work has begun to specify what encourages adoption of GenAI for health information seeking and what discourages it. Candidate influences include perceived convenience and clarity, perceived credibility and risk, privacy concerns, literacy-related resources, and interface conditions that make verification more or less feasible [9,24,25]. Yet, the evidence remains dispersed across health contexts, populations, tools, and theoretical framings, and studies often use incompatible definitions and outcomes, limiting the field’s ability to determine which factors reliably facilitate constructive use and which function as barriers that constrain use or promote inappropriate reliance [26]. This fragmentation is especially limiting because GenAI is increasingly positioned as an intermediary that shapes how health questions are formulated, which uncertainties are prioritized, and what counts as a satisfactory answer within the interaction.

Existing review-level work has examined GenAI in health care primarily from broader clinical, implementation, or ethics-oriented perspectives rather than from the perspective of health information seeking as a user practice. For example, Yim et al [27] reviewed early uses of GenAI in health care clinical services, with a focus on service delivery and clinical encounters rather than consumer or patient information seeking. Reddy [28] provided an implementation-oriented overview of GenAI in health care, emphasizing translation, integration, and governance. Wang et al [29] synthesized applications and ethical issues in mental health. Together, these reviews show growing interest in GenAI in health, but they do not specifically map how empirical studies have examined consumer and patient health information seeking through GenAI, including the roles of trust, reliance, verification, and downstream implications for clinician-patient interaction. The present scoping review addresses this gap by focusing specifically on GenAI-mediated health information seeking as a user behavior and by synthesizing facilitators, barriers, and verification-related features across this emerging literature.

A scoping review is therefore needed to consolidate empirical evidence on determinants of GenAI-mediated health information seeking, identify where findings are thin or inconsistent, and clarify how these determinants may influence verification practices and subsequent engagement with clinicians and health services.

### Objectives

Building on the gaps identified in the literature, this scoping review maps and synthesizes empirical research on the use of GenAI tools for health information seeking. As GenAI systems become more integrated into health-related decision-making, the review examines the populations, contexts, health topics, and tool types represented in the literature; maps how key outcomes and influencing factors are conceptualized and measured; and identifies gaps that should be addressed to support safe, informed use and to clarify the implications of GenAI-mediated health information for users and health care encounters. In this review, we use the term “GenAI” as the broad umbrella term for GenAI systems and “LLM-based tools” when referring more specifically to tools powered by LLMs. Terms such as “AI chatbots,” “chatbots,” and named tools (eg, ChatGPT) are used only when they reflect the terminology of the original studies or the specific interface examined. This scoping review addresses 3 research questions (RQs):

- RQ1: what study characteristics and contexts define the current literature on GenAI-based health information seeking (eg, populations, health topics, tools, and study designs)?
- RQ2: how are GenAI-based health information-seeking outcomes conceptualized and examined in the literature, including intention to use, actual use, reliance, verification, trust, and related behavioral responses?
- RQ3: what facilitators, determinants, and barriers shape users’ intention to use, adoption, or continued use of GenAI-based tools for health information seeking?

## Methods

### Protocol and Registration

This scoping review was conducted in accordance with Joanna Briggs Institute guidance for scoping reviews and is reported in line with PRISMA-ScR (Preferred Reporting Items for Systematic Reviews and Meta-Analyses extension for Scoping Reviews; Multimedia Appendix 1). The literature search was reported according to PRISMA-S (Preferred Reporting Items for Systematic Reviews and Meta-Analyses Search Extension) to improve transparency and reproducibility, with full search documentation provided in Multimedia Appendix 2 [30]. The review synthesizes empirical research examining the use of GenAI and LLM-based tools for health information seeking. The search strategy was reviewed and refined collaboratively by the authors before final execution. A review protocol was developed by the authors before data extraction.

### Eligibility Criteria

A scoping review approach was selected because research on GenAI-based health information seeking is still emerging and remains heterogeneous in terms of study designs, populations, health contexts, GenAI tools, and outcome definitions. In addition, the included studies examine a broad range of constructs, including intention to use, actual use, trust, reliance, verification, and related behavioral responses, which are not yet operationalized consistently across the literature. Accordingly, the purpose of this review was not to estimate a pooled effect but to map the scope and characteristics of the evidence, synthesize how key outcomes and influencing factors have been conceptualized, and identify gaps to guide future research and practice.

Eligibility criteria were specified a priori and applied at both title and abstract screening and full-text review. We included empirical studies (quantitative, qualitative, or mixed methods) in which users sought health-related information through a GenAI- or LLM-based system and in which the study examined health information seeking as a user behavior or investigated factors shaping that process. Eligible outcomes included intention to use, actual use, reliance, verification, source switching, escalation to clinicians, or determinants of these behaviors, such as trust, perceived credibility, perceived usefulness, literacy-related capability, privacy concerns, and perceived risk. Eligible publication types were peer-reviewed journal articles and full peer-reviewed conference papers. Conference papers were included only when they were published in established proceedings venues indexed through major publisher platforms used in the search, such as ACM Digital Library and IEEE Xplore, where the publication source identified the paper as part of formal peer-reviewed conference proceedings. Studies had to be available in full text, published in English, and published from 2022 onward.

Eligible populations included any health information seekers, including the general public, patients, caregivers, students, and health professionals, when the behavior under study involved seeking health or clinical information via a GenAI- or LLM-based system. Health topics could include symptom and triage advice seeking, conditions, treatments, medications, prevention, mental health, lifestyle and health management, and health services navigation. Eligible contexts included any setting, such as home or personal use, education, community, or clinical and telehealth support, provided that the study examined determinants or outcomes of seeking, reliance, verification, source switching, or related behavioral responses in relation to GenAI or LLM use.

Studies were considered ineligible when GenAI was evaluated only as a technical system, a clinical performance tool, or a general writing or administrative aid, without examination of health information seeking as a user behavior. We also excluded studies that assessed answer accuracy, benchmarking, or safety alone unless they also examined user-facing seeking, reliance, verification, or related behavioral outcomes. For example, we excluded studies that only compared the factual accuracy, completeness, or safety of LLM-generated health answers with clinician-generated or guideline-based answers if they did not also examine how users sought, interpreted, relied on, verified, or acted on those outputs.

Review articles, editorials, opinion pieces, conference abstracts, preprints not accepted for publication, other nonempirical papers, and non-peer-reviewed gray literature were excluded. We further excluded studies in which GenAI was used only for nonseeking tasks, such as writing, documentation, or administrative workflow, as well as studies that did not report any GenAI- or LLM-mediated health information-seeking outcome, including system-only performance outcomes or general attitudes not tied to use, reliance, verification, source switching, or related behaviors.

### Information Sources

The search strategy was developed iteratively to identify empirical studies on GenAI-mediated health information seeking across health, behavioral, education, and technology-oriented sources. In line with the scoping review design, searches were conducted in PubMed, Scopus, PsycINFO, Web of Science, IEEE Xplore, ACM Digital Library, ERIC, EBSCO, and ProQuest. Google Scholar was used as a supplementary search source. Records retrieved from Google Scholar were imported into Covidence (Veritas Health Innovation), which identified duplicate records prior to screening.

### Search

Searches combined terms related to three concept groups: (1) GenAI and LLMs, (2) health and medical contexts, and (3) health information seeking and related behavioral outcomes. Search syntax was adapted to the requirements of each source, using controlled vocabulary where available and free-text terms in titles, abstracts, and keywords. Searches were limited to English-language publications from 2022 onward, consistent with the eligibility criteria. The literature search was finalized on January 8, 2026, and identified a total of 3004 records across the selected databases.

A simplified example of the search logic was: (“generative AI” OR “large language model*” OR ChatGPT OR chatbot*) AND (health OR medical OR symptom* OR treatment* OR patient*) AND (“health information seeking” OR “information seeking” OR trust OR verification OR reliance OR “intention to use”). The full PRISMA-S search documentation, including the complete database-specific search strategies, applied limits, search dates, supplementary searching procedures, record management, deduplication procedures, and items that were not applicable or not conducted, is provided in Multimedia Appendix 2.

### Selection of Sources of Evidence

All retrieved records were imported into Covidence for automated deduplication and screening workflow management. Screening proceeded in sequential stages: (1) pilot calibration of eligibility criteria on an initial subset of records to harmonize interpretations, (2) independent title and abstract screening by 2 reviewers against the inclusion criteria, and (3) independent full-text review of potentially eligible articles by both reviewers. Interrater reliability was assessed using Cohen κ, yielding κ=0.86, indicating strong agreement between reviewers. At the full-text stage, reasons for exclusion were recorded systematically within Covidence to maintain an auditable decision trail. Disagreements at the title and abstract or full-text screening stages were resolved through discussion between the 2 reviewers until consensus was reached.

### Data Charting Process

A structured data extraction form was developed and piloted prior to full extraction to ensure consistent interpretation of the review constructs. For each included study, we charted bibliographic details, publication year, country and setting, study design and methods, population characteristics, sample size, health topic or context, and the GenAI- or LLM-based tool and interaction modality examined. We also extracted the main behavioral outcomes and determinants addressed in the study, including intention to use, actual use, reliance, verification, source switching, escalation to clinicians, trust, perceived credibility, perceived usefulness, privacy concerns, and literacy-related capability. In addition, we charted the facilitators, barriers, and verification-supporting features reported across studies. Data extraction was conducted by 1 author and reviewed by the second author for accuracy and consistency, with disagreements resolved through discussion.

### Data Items

The main data items charted from each study were bibliographic information, publication year, population, health topic or domain, study design, sample size, GenAI- or LLM-based tool examined, key findings, and reported factors shaping use. Reported factors were further categorized as facilitators, barriers, limiting conditions, capability-related factors, and verification-supporting features when these were described in the included studies. Population type, health domain, outcome construct, facilitator, barrier, and verification-supporting feature were treated as non–mutually exclusive categories when studies addressed more than 1 relevant construct. When terminology differed across studies, closely related constructs were grouped under broader review-level categories while preserving the meaning of the original findings. The full findings table is provided in Multimedia Appendix 3.

### Synthesis of Results

The included studies were heterogeneous in design, population, health context, GenAI tool, and outcome definition; hence, the charted data were synthesized descriptively and narratively. We first summarized study characteristics using frequencies and proportions for study design, population type, country or region, health context, GenAI- or LLM-based tool, interaction modality, and sample size. These summaries were used to map the scope and distribution of the evidence base in relation to RQ1.

For RQ2, reported outcomes were grouped into conceptually distinct categories, including intention or willingness to use, actual or self-reported use, reliance, trust or credibility, verification behavior, preference or switching, and downstream action. These categories were developed from the review questions and refined during data charting when studies used overlapping or inconsistent terminology. Studies that reported more than 1 relevant outcome were coded into all applicable categories.

For RQ3, facilitators, barriers, and verification-related factors were synthesized using an iterative narrative approach. Factors were first extracted using the terminology of the included studies and then grouped into higher-level categories, including efficiency and access, comprehensibility and presentation quality, personalization and specificity, affective or interpersonal comfort, credibility and trust concerns, privacy or data security concerns, perceived unsuitability for complex or high-stakes situations, prompting- or literacy-related capability, and modality or interaction constraints. Categories were not mutually exclusive, and individual studies could contribute to more than 1 category. Frequencies and percentages were calculated to describe how often each factor was reported across the included studies, but these counts were used to map patterns in the evidence base rather than estimate effects.

Finally, an evidence gap map was constructed by cross-tabulating health domains against outcome constructs. This map was used to identify areas where evidence was concentrated and areas where empirical evidence was limited or absent. The synthesis was organized around the 3 RQs and focused on mapping the range, distribution, and conceptual structure of the evidence.

## Results

### Selection of Sources of Evidence

**Figure 1:**
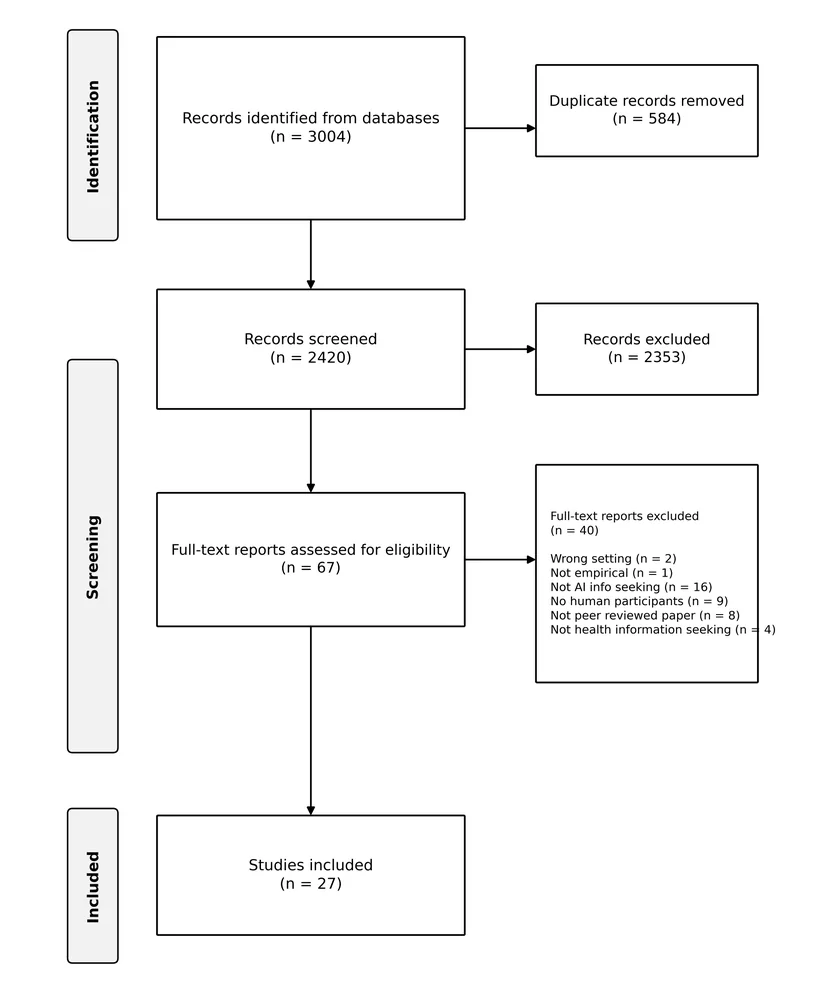
PRISMA (Preferred Reporting Items for Systematic Reviews and Meta-Analyses) 2020 flow diagram of study selection for this scoping review of generative AI (GenAI)–mediated health information seeking. The diagram shows records identified, screened, excluded, and included from searches conducted from 2022 onward.

The literature search identified 3004 records across the selected databases. After removing 584 duplicates, 2420 unique records remained for title and abstract screening. We assessed 67 full-text articles for eligibility. Following full-text review, 40 articles were excluded for prespecified reasons. The most common reasons were not AI information seeking (n=16), no human participants (n=9), and not peer-reviewed papers (n=8). Additional exclusions reflected not health information seeking (n=4), the wrong setting (n=2), and not empirical (n=1). In total, 27 studies met the inclusion criteria and were included in this review (Figure 1).

### Characteristics of Sources of Evidence

We first examined the characteristics of the included studies to describe the evidence base in terms of study designs, populations, health contexts, and the GenAI tools examined (Table 1). Most studies used survey designs (n=11; [1,5,9,11,20-24,26,31]), followed by qualitative designs (n=10; [7,12,13,25,32-37]), with fewer mixed methods studies (n=4; [2,6,38,39]) and relatively few experimental designs (n=2; [40,41]).

**Table 1:** Distribution of the 27 included studies by study design and health-topic focus. Studies covered general health information seeking as well as condition-specific contexts, including cancer, mental health, chronic conditions, and care navigation.

Characteristic		Studies, n (%)
**Study design**		
	Cross-sectional survey	11 (40.7)
	Qualitative	10 (37)
	Mixed methods	4 (14.8)
	Experimental	2 (7.4)
**Health-topic focus**		
	General health information seeking	15 (55.6)
	Condition or pathway-specific	12 (44.4)

In terms of participants, more than half of the studies focused on general adult or general public samples (n=15). Clinical or care-related samples, including patients and caregivers in condition- or pathway-specific contexts, were represented in a smaller set (n=6). Student samples appeared in 3 studies, and 3 studies focused on specific community groups, including older Black or African American adults and underserved populations. One study examined clinicians’ perspectives.

In terms of topic focus, many studies examined GenAI use for health information seeking in general terms, such as everyday health questions, symptom interpretation and advice seeking, or health-related searching without anchoring the analysis in a single condition or care pathway (n=15). The remaining studies were situated in defined domains or care situations (n=12), including cancer-related information seeking and treatment decision support [34,37], chronic condition management [35], perioperative or procedure-related information needs, musculoskeletal pain (eg, low back pain) [24,32], gastrointestinal conditions (eg, irritable bowel syndrome) [11], pediatric subspecialty contexts (eg, rheumatology) [39], cognitive disorders (eg, Alzheimer disease and related dementias) [12], sexual health [22,33], and mental health help-seeking [28,38].

Finally, general-purpose LLM chat tools were the most frequently examined systems (n=12; [5-7,9,21,24,26,31,34-36,39]), whereas the remainder examined GenAI broadly as a category of tools (n=15). A subset explicitly examined interaction modality (n=4; [6,12,22,42]), including comparisons involving text chat, voice assistants, mobile or app-based interactions, and embodied agents, while most studies treated text-based chatbot interaction as the default mode.

To provide a more comprehensive picture of the evidence base, we examined the geographic distribution and methodological characteristics of the included studies in greater detail. The largest share of studies was conducted in the United States (n=8), followed by Saudi Arabia (n=3), the United Kingdom (n=3), and China (n=3). Two studies were conducted in Jordan and 2 in Hong Kong. Single studies came from Nigeria, Australia, Germany, Hungary, the Czech Republic, and 1 multicountry study. This distribution indicates a concentration of evidence from English-speaking and high-income settings, with limited representation from low- and middle-income countries, sub-Saharan Africa, Latin America, and South and Southeast Asia.

**Figure 2:**
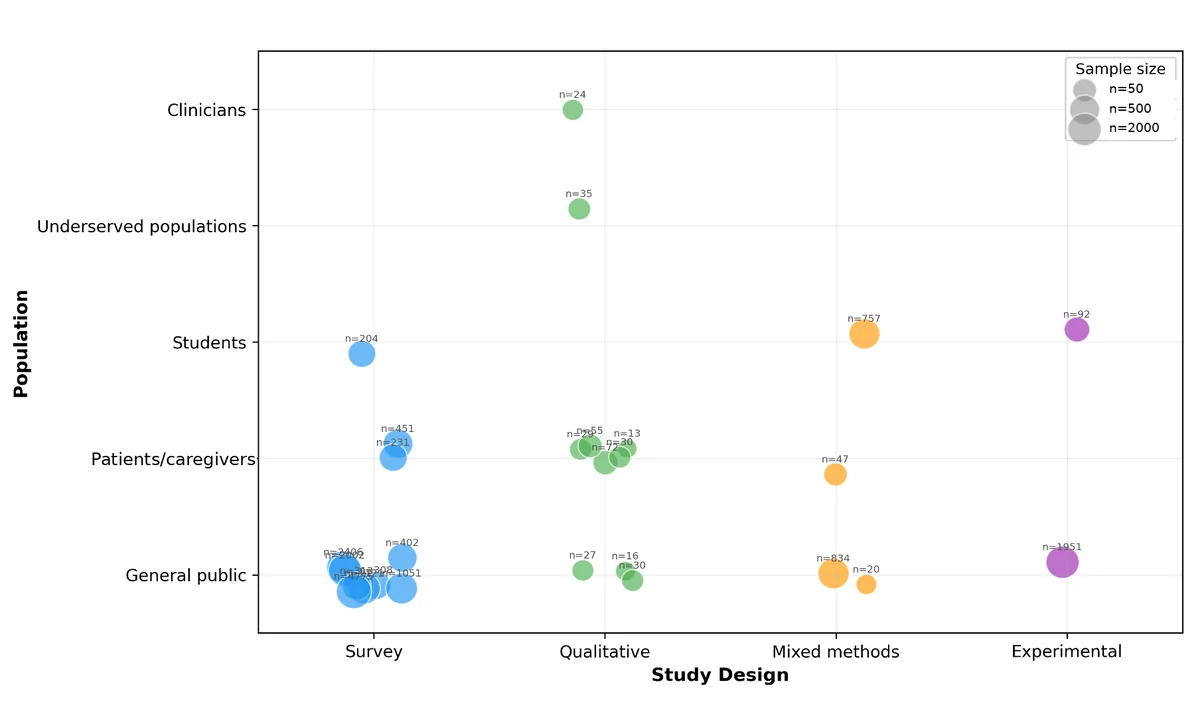
Distribution of the 27 included studies by study design, population type, and sample size. Each bubble represents 1 study, with bubble size proportional to the logarithm of the sample size. Surveys dominated the evidence base with larger samples, while qualitative designs were more common for patient-focused investigations.

Figure 2 further illustrates the distribution of studies by study design, population, and sample size. Surveys tended to recruit larger samples (median approximately 400 participants), whereas qualitative studies typically involved smaller groups (median approximately 30 participants). The general public was the most frequently studied population across all designs, whereas patients and caregivers were more often examined through qualitative approaches. Student and clinician populations were examined in only a small number of studies. The 2 experimental studies were notably few relative to the overall corpus, and both involved nonclinical populations.

### Synthesis of Results

Across the included studies, outcome constructs were heterogeneous and should not be treated as interchangeable. In particular, intention to use, actual use, reliance, verification behavior, source switching, and downstream actions represent distinct behavioral constructs and were examined unevenly across the literature.

Most studies operationalized adoption-related outcomes as intention, willingness, or preference rather than as observed behavior. Intention to use or willingness to use GenAI for health information was the most common outcome type [1,2,5-7,9,11-13,20-23,25,26,33,40,41], and several studies supplemented these measures with comparative preference judgments between GenAI tools and other information channels [24,31,32,34,35]. This suggests that much of the current evidence reflects anticipated or self-reported openness to use rather than demonstrated reliance in practice.

A smaller subset of studies examined actual or self-reported use. Where prevalence was measured, GenAI use for health queries appeared meaningful but generally remained secondary to conventional search. For example, 1 large US survey found that 21.5% of respondents had used ChatGPT for health information, and among those users, 39.3% reported doing so 2 to 3 times per week [21]. In another survey comparing channels, search engines were used more often than LLMs for health queries and were also more likely to be the first source consulted [5]. Similarly, in a condition-specific survey of patients with irritable bowel syndrome, AI chatbot use for health information was reported less often than the use of search engines and official health websites [11].

Reliance-related outcomes were less often measured directly, but several studies suggested that GenAI was typically used as part of a broader information routine rather than as a replacement for other sources [5,7,12,32,33]. Across studies that described what GenAI was used for, the most common tasks involved early-stage orientation and decision support [25,32,34,36,38-40]. These uses included symptom interpretation, deciding whether professional consultation might be needed, clarifying terminology, and exploring options or alternative treatments. In these studies, users often began with a conversational query and then shifted to other channels when they wanted stronger credibility cues, inspectable sources, or greater confidence in the information.

Verification behavior was also examined unevenly across the included studies and was conceptually distinct from both use and reliance. In several studies, users described checking GenAI outputs against other sources, comparing responses across queries, or seeking confirmation from clinicians or trusted websites when the stakes were higher or credibility was uncertain [7,12,13,32,33]. These findings suggest that verification was not a routine or standardized outcome across the literature but rather an episodic practice shaped by perceived risk, source visibility, and the availability of alternative credibility cues.

Switching and downstream actions were examined much less often. One study explicitly operationalized intention to switch from traditional online health information platforms to GenAI as its focal adoption-related outcome [2]. Only 1 study reported concrete downstream actions after GenAI use for health information, including bringing AI-generated information to a physician, requesting a referral or test, or taking medication [21]. These findings suggest that downstream behavioral consequences remain underexamined in the current evidence base.

Overall, the literature more often conceptualized adoption in terms of intention to use than observed or behaviorally anchored use. Taken together, the findings suggest that GenAI and LLM tools are currently more often used to complement conventional search and official sources than to replace them.

**Figure 3:**
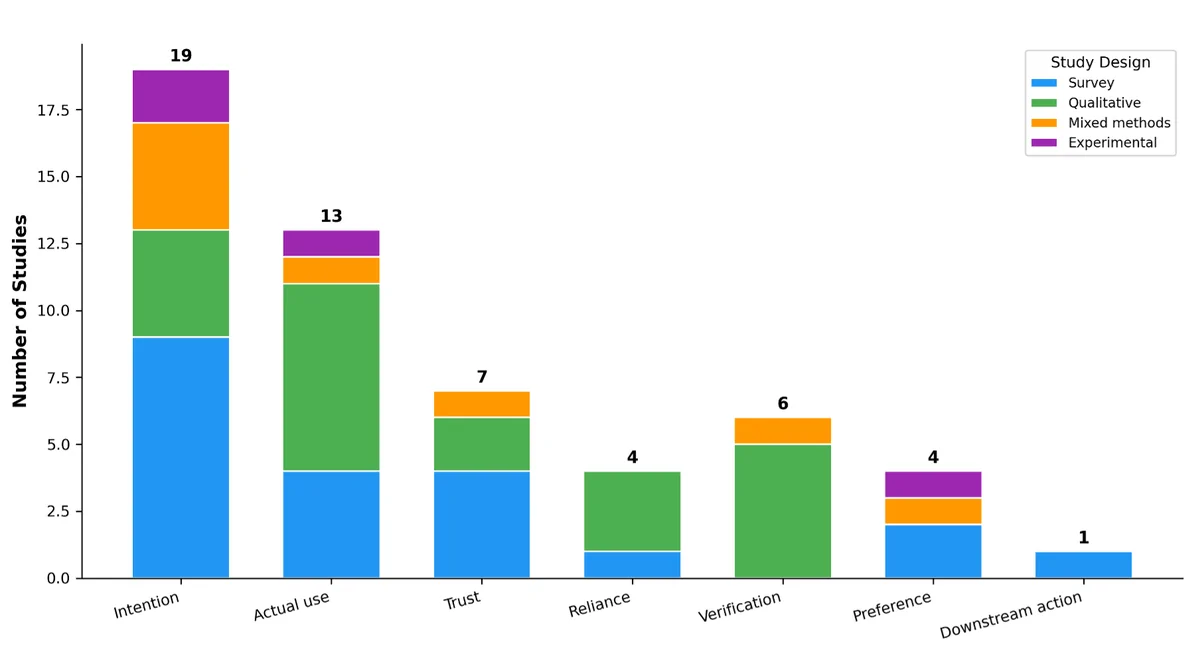
Number of studies examining each outcome construct, stratified by study design (n=27). Categories are not mutually exclusive, as individual studies could examine more than 1 outcome. Intention to use was the most commonly studied outcome, while downstream action was examined in only 1 study.

Figure 3 provides a visual summary of how different outcome constructs were distributed across study designs. Intention to use was the most commonly examined outcome, reported in 19 of 27 studies, and was predominantly assessed through survey designs. Actual use was examined in 13 studies, drawing on both surveys and qualitative investigations. Trust and credibility were the focus of 7 studies, reliance was examined in 4, and verification behavior in 6. Preference or switching intention appeared in 4 studies, while downstream action, such as bringing GenAI-generated information to a clinician, was examined in only 1 study. This imbalance indicates that the literature remains heavily focused on early-stage adoption constructs, with substantially less attention to the behavioral consequences of GenAI use in real-world health contexts.

To synthesize factors shaping GenAI-mediated health information seeking, we focused on constructs described as enabling or constraining use during health information-seeking episodes (Table 2).

**Table 2:** Facilitators, barriers, and capability-related factors shaping GenAI-mediated health information seeking across the 27 included studies. Percentages indicate the proportion of studies reporting each factor, and categories are not mutually exclusive.

Characteristic		Studies, n (%)
**Facilitators**		
	Comprehensibility and presentation quality	11 (40.7)
	Efficiency and access	8 (29.6)
	Affective and interpersonal comfort	5 (18.5)
	Personalization and specificity	5 (18.5)
	Human-like interaction and lower negative affect	3 (11.1)
**Barriers**		
	Credibility and trust concerns	13 (48.1)
	Modality and interaction constraints	5 (18.5)
	Unsuitable for complex, urgent, or emotional situations	5 (18.5)
	Privacy and data security concerns	4 (14.8)
	Limited prompting skills	2 (7.4)

Facilitators largely reflected lower friction and better comprehensibility. Several studies (n=8) described efficiency and access, including reduced time and effort, support between appointments, and around-the-clock availability [12,32-34,37-39,41]. Other studies (n=11) highlighted comprehensibility and presentation quality, with users valuing simpler language, clearer explanations, and more direct guidance than link-based search [2,7,11-13,22,24,25,33,36,41]. Several studies (n=5) also emphasized personalization and specificity, especially when responses were tailored to a condition or situation or when users prompted for more contextual detail [6,9,32,37,39]. Five studies also described affective and interpersonal comfort, including less embarrassment when asking about sensitive topics, a sense of nonjudgmental interaction, and the option to remain anonymous [5,22,24,32,38]. In 3 studies, these benefits extended to a more human-like interaction style and lower negative affect during use [5,24,38].

Barriers were dominated by trust and perceived risk. Several studies (n=13) raised credibility and trust concerns, particularly when cues for evaluating accuracy were missing or hard to interpret [9,11,13,21-24,27,29,31,43-45]. In those cases, users questioned whether responses were correct, were concerned about absent or unreliable citations, and were reluctant to treat GenAI as a stand-in for clinical expertise when the stakes were higher. Four studies described privacy and data security concerns [6,35,45,46]. Five studies emphasized that GenAI felt poorly suited to complex, urgent, or emotionally charged situations, including worries about miscommunication and a preference for human contact or clear escalation routes [21,24,28,44,45]. Practical constraints also appeared, with 2 studies noting limited prompting skills as a barrier to effective use [26,27]. Five studies pointed to modality and interaction constraints that made it harder to retain information, compare claims, or judge credibility [6,7,13,25,35].

Uncertainty management depended on users’ capabilities and interaction skills, with several studies (n=9) indicating that familiarity [9,23,34], digital proficiency and literacy [5,21,23,30,35,44,46], and topic knowledge [26] shaped how well users could evaluate outputs. In 5 studies [6,7,13,25,35], evaluation of outputs felt more manageable when tools provided concrete verification and reuse affordances, such as visible sourcing, accessible transcripts, and options to save, revisit, or share content. When credibility cues were available, such as a sense of reliability, inspectable sourcing, or signals of professional legitimacy, confidence tended to increase. Overall, the evidence suggests that convenience and clarity encourage use, whereas concerns about credibility, safety, and privacy constrain it, especially when the perceived stakes are high.

**Figure 4:**
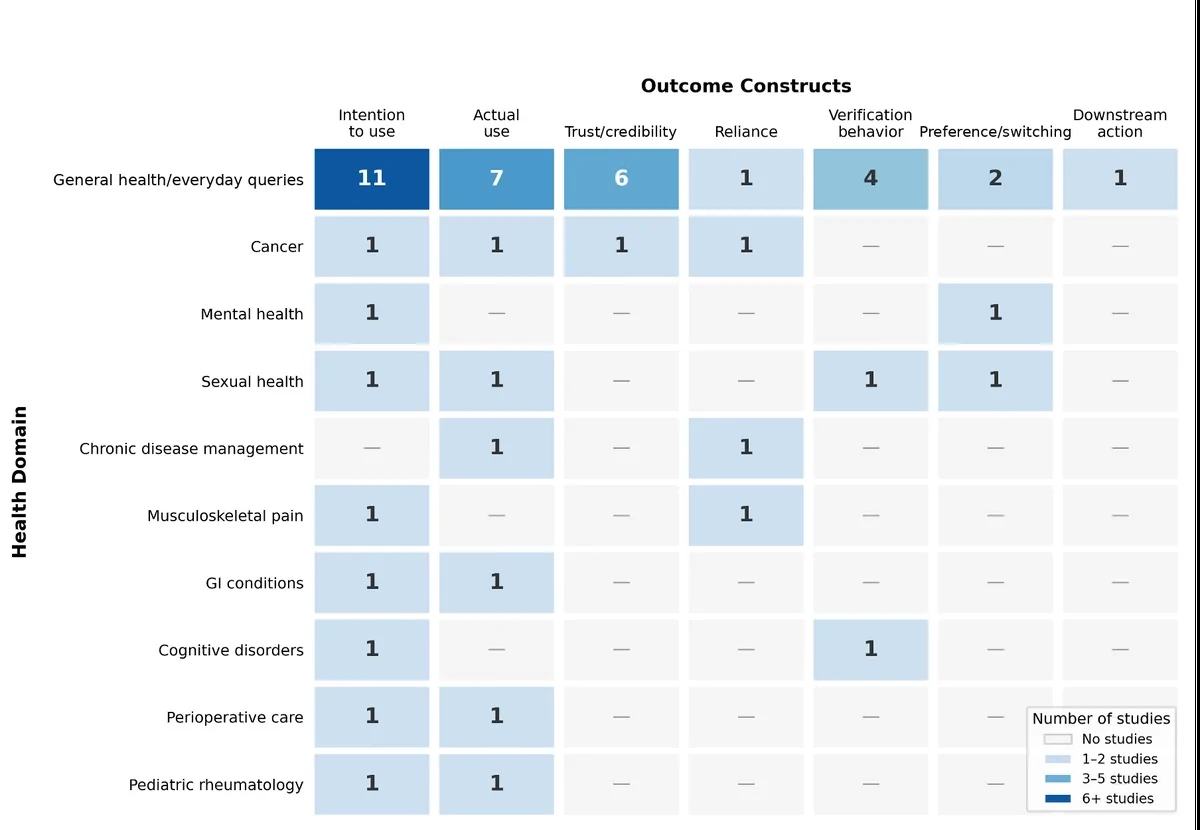
Evidence gap map cross-tabulating health domains against outcome constructs across the 27 included studies. Cell values indicate the number of studies addressing each domain-outcome combination. Darker shading indicates greater evidence concentration, while dashes indicate no available studies. The map reveals that most evidence is concentrated in the general health domain and on intention-related outcomes, with substantial gaps in condition-specific contexts and in behavioral outcomes such as reliance, verification, and downstream action. GI: gastrointestinal.

To identify where evidence is concentrated and where gaps remain, we constructed an evidence gap map (Figure 4) that cross-tabulates the health domains examined across studies against the outcome constructs reported. The gap map reveals that the general health and everyday queries domain is the most densely populated, with studies addressing intention (n=11), actual use (n=7), trust (n=6), and verification behavior (n=4). In contrast, condition-specific domains such as cancer, mental health, sexual health, chronic disease management, musculoskeletal pain, gastrointestinal conditions, cognitive disorders, perioperative care, and pediatric rheumatology were each represented by only 1 or 2 studies per outcome construct. Several cells in the map contain no studies at all, indicating that many combinations of health domain and outcome construct remain entirely unexamined.

The gap map also highlights that reliance, verification behavior, preference or switching, and downstream action are understudied across nearly all health domains. For example, downstream action was examined in only 1 study, and only within the general health domain. Verification behavior was addressed in the general health, sexual health, and cognitive disorders domains but not in cancer, chronic disease, musculoskeletal, gastrointestinal, perioperative, or pediatric contexts. These gaps suggest that the current evidence base is insufficient to draw conclusions about how trust, reliance, and verification operate in condition-specific or high-stakes health scenarios, where the consequences of acting on inaccurate information may be most serious.

## Discussion

### Summary of Evidence

This scoping review examined what facilitates and constrains GenAI use for health information seeking and how trust, verification practices, and perceived stakes shape that use. Across the included studies, GenAI was rarely treated as a replacement for established health information pathways [5,11,13,21-23]. Instead, it more often functioned as an initial orientation layer that supported symptom interpretation, clarification, and option exploration, while higher-stakes situations prompted checking, source comparison, or escalation to clinicians. Overall, the findings suggest that GenAI-mediated health information seeking is shaped not only by convenience and clarity but also by users’ ability to assess credibility, judge risk, and decide when AI-generated information is sufficient and when it requires verification or professional follow-up.

A central pattern across the literature is that GenAI use is strongly contingent on perceived stakes. When users viewed a situation as lower stakes, GenAI was often treated as a convenient and accessible tool for preliminary understanding or sensemaking [7,13,21,24,32,34,36]. As perceived stakes increased, however, users were more likely to seek stronger credibility anchors through clinician consultation, trusted websites, or other external sources [13,21-24,32,36]. This suggests that GenAI may be incorporated into a tiered health information ecology, in which the same system may be experienced as useful in one context and inappropriate in another, depending on urgency, emotional load, and the perceived consequences of error [5,7,11-13,24,32,33,36].

The review also suggests that trust in GenAI-mediated health information is not static but develops within the interaction itself as users interpret the system’s cues, weigh the stakes of the situation, and decide whether the response is credible enough to act on or should be verified elsewhere [6,9,20,23,24,26]. In this sense, adoption and continued use may depend less on generalized positive attitudes toward AI and more on whether the interaction reduces uncertainty while supporting credibility assessment in practice. This point is important because miscalibration can operate in 2 directions: users may place too much confidence in outputs that do not warrant it, or they may dismiss tools that could still serve as useful support in lower-stakes situations [21,23,24,36]. The findings therefore point to the importance of designing for warranted trust by making credibility assessment easier during use, including through visible sourcing, interpretable uncertainty cues, and interface features that support comparison, revisiting, and sharing [6,7,12,13,25,45].

Overall, the findings suggest an integrative process in which GenAI-mediated health information seeking unfolds through 4 linked mechanisms: reduced access costs, altered credibility evaluation, dynamic uncertainty management, and conditional escalation. GenAI appears to lower the effort required to obtain a coherent answer, but this convenience shifts more credibility work to the user, especially when sourcing and uncertainty cues are weak [6,7,12,13,25,32,36]. In this process, verification is shaped not only by trust in the tool, but also by perceived stakes, available alternatives, and the interactional features of the interface [6,7,12,13,21,32]. This positions GenAI not simply as another information source but as a distinct information environment that may reconfigure how users assess, verify, and carry information into subsequent clinical encounters [4,5,7,13,21].

These findings suggest implications for clinician-patient interaction because GenAI can shape both the information patients encounter and the form in which that information enters the clinical visit [4,21-23]. Rather than arriving with a set of disconnected search results, patients may come with a synthesized narrative that already combines explanation, interpretation, and implied recommendations [4,10,21]. This can influence agenda setting, expectations, and the tone of the encounter, particularly when the generated explanation feels coherent and persuasive or when it conflicts with previous clinical advice [4,10,21]. Existing evidence suggests that AI-generated health information is sometimes acted on and can affect patient-provider dynamics, which makes it clinically relevant to ask not only what information patients have consulted, but also whether GenAI tools shaped their understanding before the visit [21-23].

One practical implication is the value of brief, repeatable routines that support calibration and shared decision-making [4,7,21-23]. Clinicians can ask what tool was used, what the patient asked, whether sources were provided, and what forms of verification were attempted. Such routines may help distinguish lower-stakes sensemaking from situations in which reliance could be harmful while also translating the GenAI output into clinically meaningful next steps, including what seems plausible, what remains uncertain, and what requires examination or testing [4,7,21]. This is especially relevant because credibility assessment in AI-assisted health information seeking does not fully mirror traditional web search; users appear to rely on different cues, and these are shaped in part by interface-level affordances [7,13].

Privacy and security considerations add a further layer of complexity to the integration of GenAI into health information practices [44]. Conversational querying may invite users to disclose sensitive details in pursuit of more tailored responses; yet, the included studies suggest uneven awareness of downstream privacy risks [6,35-37,44,46]. Privacy concerns can therefore function both as a barrier to use and as a factor shaping what users are willing to disclose and how they approach verification [6,35,36,44]. In practical terms, this supports the value of brief privacy-aware guidance, such as avoiding identifiable details, using GenAI for general education or question preparation rather than diagnosis, and escalating urgent or high-stakes concerns through clinical channels [35,37,44,46].

The findings also point to clear design implications. If users are expected to engage in verification, GenAI systems should make that process straightforward rather than burdensome [6,7,12,13,25]. This includes surfacing sources in ways that are easy to inspect, differentiating between evidence-based content and model-generated inference, and signaling uncertainty or limits when questions are high stakes or outside the system’s reliable scope [13,25]. Interfaces may also support safer use by enabling comparison and reuse through persistent transcripts, save and revisit functions, and sharing features that help users bring AI-generated content into discussion with clinicians or others [6,7,12,13,25]. More broadly, design can support better calibration by prompting escalation when urgency cues appear, encouraging more specific follow-up questioning without eliciting unnecessary identifiers, and embedding privacy-protective defaults into the interaction [6,7,13,25,44-46].

Several research priorities follow from this synthesis. First, future studies should distinguish more clearly among intention to use, actual use, reliance, verification behavior, and downstream action, as these reflect different forms of engagement and risk [2,5,7,21,23,24,42]. Second, research should test whether visible sourcing, uncertainty cues, and transcript-based comparison tools improve calibration and reduce inappropriate reliance [6,7,12,13,25,45]. Third, more work is needed on how GenAI-mediated information seeking enters clinician-patient interaction, including whether AI-shaped interpretations influence agenda setting, expectations, and follow-up decisions [4,21-23]. Fourth, studies should examine these processes in higher-stakes and underserved contexts, where verification burdens, literacy demands, and access constraints may be especially consequential [12,21,23,25,32,33,37].

Some of the variation across findings likely reflects differences in what studies were actually measuring and in the contexts in which GenAI was used. In several cases, studies used related but nonequivalent constructs, such as intention to use, trust, reliance, verification, or actual use, which makes direct comparison difficult [2,5-7,21,23,24,42]. Variation may also reflect differences in population, as members of the general public, patients, students, and clinicians may approach these tools with different expectations, needs, and thresholds for risk [12,21,22,25,29,31,37]. In addition, not all studies examined the same kinds of systems: some focused on chatbot-style interfaces, others on named tools such as ChatGPT, and not all tools offered the same sourcing, interaction, or comparison features [6,7,12,13,25,35]. Context may matter as well, since lower-stakes information seeking is likely to produce different patterns of trust and use than situations involving urgency, complexity, or emotional strain. Rather than treating these findings as simply inconsistent, it may be more accurate to understand them as context-sensitive patterns within an emerging evidence base.

### Limitations

This review has several limitations, and its conclusions should be interpreted cautiously because the evidence base remains relatively small and methodologically heterogeneous, with many studies relying on self-reported intentions, attitudes, and perceptions rather than observed behavior. First, heterogeneity in study designs, populations, tools, and outcomes limited direct comparison across studies. Second, key constructs such as intention, use, reliance, trust, and verification were operationalized inconsistently, making it difficult to compare findings across studies and to distinguish clearly among adjacent concepts such as adoption and reliance. Third, reporting of tool identity, model version, interface features, and prompting conditions was often incomplete, which reduced reproducibility and limited interpretation of which system characteristics may have shaped observed outcomes. Fourth, many studies focused on lower-stakes or relatively controlled contexts, with less attention to acute, complex, or emotionally charged situations in which risks and escalation decisions may be more consequential. Finally, differences in health literacy, digital literacy, and access are likely to shape both use and verification capacity; yet, subgroup analyses were not consistently reported, limiting equity-oriented interpretation.

Overall, this review highlights the need to understand GenAI in health not only as a technical system but as an emerging information environment through which users seek, interpret, and act on health-related information. Its contribution lies in suggesting that the benefits and risks of GenAI-mediated health information seeking are shaped not only by model quality but also by how trust is calibrated, how verification is supported, and how users navigate uncertainty and perceived risk. In broader real-world terms, improving safety in this area may require more than better outputs; it may also require interface designs, educational supports, and clinical communication practices that help people use AI-generated health information in ways that are informed, proportionate, and appropriately verified.

### Conclusions

This review is innovative in reframing GenAI in health not primarily as a clinical decision tool but as an emerging information environment through which users seek, interpret, and act on health-related information. Unlike prior reviews that have focused mainly on technical performance, diagnostic accuracy, or clinical implementation, the present review synthesizes empirical evidence on health information seeking as a user practice and clarifies how the field has conceptualized use, trust, reliance, and verification. In doing so, it provides a clearer map of the facilitators, barriers, literacy-related capabilities, and verification-supporting features that shape GenAI-mediated health information behavior across a still-fragmented literature. Its real-world implication is that improving safety in this domain requires more than better models; it also requires interfaces, educational supports, and clinical communication strategies that help users evaluate and appropriately act on AI-generated health information.

## Appendix

Multimedia Appendix 1 PRISMA-ScR checklist.

Multimedia Appendix 2 PRISMA-S search documentation and database-specific search strategies.

Multimedia Appendix 3 Characteristics and charted findings of included studies.

## Abbreviations

GenAI: generative AI
LLM: large language model
PRISMA-S: Preferred Reporting Items for Systematic Reviews and Meta-Analyses Search Extension
PRISMA-ScR: Preferred Reporting Items for Systematic Reviews and Meta-Analyses extension for Scoping Reviews
RQ: research question
