# Supplementary material for: Consumer and Patient Health Information Seeking With Generative AI Tools: Scoping Review of Facilitators and Barriers
Source: J Med Internet Res. 2026 Sep 2;28:e93944. doi: 10.2196/93944 (PMC13583531; doi:10.2196/93944)
Supplement: Supplementary file 1 [file jmir_v28i1e93944_app1.docx]

*Multimedia Appendix 1: PRISMA-ScR Checklist*

| Section | Item | PRISMA-ScR checklist item | Reported in manuscript |
| --- | --- | --- | --- |
| Title | 1 | Identify the report as a scoping review. | Title |
| Abstract | 2 | Provide a structured summary that includes, as applicable, background, objectives, eligibility criteria, sources of evidence, charting methods, results, and conclusions that relate to the review questions and objectives. | Abstract: Background, Objective, Methods, Results, Conclusions |
| Introduction: Rationale | 3 | Describe the rationale for the review in the context of what is already known. Explain why the review questions/objectives lend themselves to a scoping review approach. | Introduction: Rationale |
| Introduction: Objectives | 4 | Provide an explicit statement of the questions and objectives being addressed with reference to their key elements. | Introduction: Objectives |
| Methods: Protocol and registration | 5 | Indicate whether a review protocol exists; state if and where it can be accessed; and, if available, provide registration information. | Methods: Protocol and registration |
| Methods: Eligibility criteria | 6 | Specify characteristics of the sources of evidence used as eligibility criteria, such as years considered, language, and publication status, and provide a rationale. | Methods: Eligibility criteria |
| Methods: Information sources | 7 | Describe all information sources in the search, as well as the date the most recent search was executed. | Methods: Information sources; Search |
| Methods: Search | 8 | Present the full electronic search strategy for at least one database, including any limits used, such that it could be repeated. | Methods: Search; Multimedia Appendix 2 |
| Methods: Selection of sources of evidence | 9 | State the process for selecting sources of evidence included in the scoping review. | Methods: Selection of sources of evidence |
| Methods: Data charting process | 10 | Describe the methods of charting data from the included sources of evidence, including whether forms were tested and whether data charting was independent or duplicate. | Methods: Data charting process |
| Methods: Data items | 11 | List and define all variables for which data were sought and any assumptions and simplifications made. | Methods: Data items |
| Methods: Critical appraisal of individual sources of evidence | 12 | If done, provide a rationale for conducting a critical appraisal of included sources of evidence; describe the methods used and how this information was used in synthesis. | Critical appraisal of individual sources of evidence was not conducted because the purpose of this scoping review was to map the scope, characteristics, and conceptual structure of the emerging evidence rather than to assess study quality, estimate effectiveness, or produce pooled effects. |
| Methods: Synthesis of results | 13 | Describe the methods of handling and summarizing the data that were charted. | Methods: Synthesis of results |
| Results: Selection of sources of evidence | 14 | Give numbers of sources screened, assessed for eligibility, and included in the review, with reasons for exclusions at each stage, ideally using a flow diagram. | Results: Selection of sources of evidence; Figure 1 |
| Results: Characteristics of sources of evidence | 15 | For each source of evidence, present characteristics for which data were charted and provide citations. | Results: Characteristics of included studies; Table 1 |
| Results: Critical appraisal within sources of evidence | 16 | If done, present data on critical appraisal of included sources of evidence. | N/A |
| Results: Results of individual sources of evidence | 17 | For each included source of evidence, present the relevant data that were charted and that relate to the review questions and objectives. | Table 1; Multimedia Appendix 3 |
| Results: Synthesis of results | 18 | Summarize and/or present the charting results as they relate to the review questions and objectives. | Results: Synthesis of results; Figures 2–7 |
| Discussion: Summary of evidence | 19 | Summarize the main results, including an overview of concepts, themes, and types of evidence available; link to the review questions and objectives; and consider relevance to key groups. | Discussion: Summary of evidence |
| Discussion: Limitations | 20 | Discuss the limitations of the scoping review process. | Discussion: Limitations |
| Discussion: Conclusions | 21 | Provide a general interpretation of the results with respect to the review questions and objectives, as well as potential implications and/or next steps. | Conclusions |
| Funding | 22 | Describe sources of funding for the included sources of evidence, as well as sources of funding for the scoping review. Describe the role of the funders of the scoping review. | The authors declared no financial support was received for this scoping review. Funding sources for the included studies were not extracted or synthesized because study funding was not part of the review questions or data-charting framework. |
