# Supplementary material for: Consumer and Patient Health Information Seeking With Generative AI Tools: Scoping Review of Facilitators and Barriers
Source: J Med Internet Res. 2026 Sep 2;28:e93944. doi: 10.2196/93944 (PMC13583531; doi:10.2196/93944)
Supplement: Supplementary file 2 [file jmir_v28i1e93944_app2.docx]

***Multimedia Appendix 2. PRISMA-S Search Documentation and Database-Specific Search Strategies***

This appendix reports the literature search according to PRISMA-S. It provides the information sources searched, complete database-specific search strategies, limits and restrictions, final search date, supplementary citation searching, record management, and deduplication procedures. Items that were not part of the search methodology are explicitly reported as not applicable or not conducted.

Searches were conducted in PubMed, Scopus, PsycINFO, Web of Science Core Collection, IEEE Xplore, ACM Digital Library, Google Scholar, ERIC, EBSCO, and ProQuest. Searches were limited to English-language publications from 2022 onward. The final updated search was run on January 8, 2026. Search syntax was adapted to the indexing structure and search interface of each source. Backward citation searching was conducted by manually examining the reference lists of included studies and relevant review papers. All retrieved records were imported into Covidence, where duplicates were removed before screening.

No study registries were searched because the review focused on peer-reviewed empirical studies. No authors, experts, or manufacturers were contacted to identify additional records. No automated search alerts were used. No formal PRESS or librarian peer review of the search strategy was conducted; however, the strategy was reviewed and refined collaboratively by the authors before final execution. No published search filter was used. No complete search strategy from a prior review was reused or adapted. Table A1 maps the search methods used in this review onto the PRISMA-S checklist items.

**Table A1.** PRISMA-S Search Reporting Checklist

| PRISMA-S item | Reporting in this review |
| --- | --- |
| Database name | PubMed, Scopus, PsycINFO, Web of Science Core Collection, IEEE Xplore, ACM Digital Library, Google Scholar, ERIC, EBSCO, and ProQuest were searched. |
| Multi-database searching | No multi-database search was conducted unless separately specified by the database platform. |
| Study registries | No study registries were searched. |
| Online resources and browsing | Google Scholar was searched as a supplementary source. |
| Citation searching | Backward citation searching was conducted by examining reference lists of included studies and relevant review papers. Forward citation searching was not conducted. |
| Contacts | No authors, experts, manufacturers, or other external contacts were approached. |
| Other methods | No additional search methods were used beyond database searching, Google Scholar, and backward citation searching. |
| Full search strategies | The exact search strategy for each database/source is provided below. |
| Limits and restrictions | Searches were limited to English-language publications from 2022 onward. |
| Search filters | English language; publication years 2022–2026. |
| Prior work | No complete search strategy from a prior review was reused or adapted. |
| Updates | The final updated search was run on January 8, 2026. No automated alerts were used. |
| Dates of searches | The final search date was January 8, 2026. |
| Peer review | No formal PRESS or librarian peer review was conducted; the strategy was reviewed and refined collaboratively by the authors. |
| Total records | The searches identified 3004 records in total. If available, source-specific counts are reported below. |
| Deduplication | Records were imported into Covidence, where duplicates were removed before screening. |

**Full database-specific search strategies**

The following search strategies are reported exactly as run in each database or supplementary source, with database-specific syntax and applied limits retained. The final updated search was conducted on January 8, 2026.

**PubMed**

Search date: January 8, 2026
Platform: PubMed/National Library of Medicine
Limits: English; publication date from January 1, 2022 onward
Search strategy:

(
 ("Artificial Intelligence"[Mesh] OR "generative AI"[Title/Abstract] OR "generative artificial intelligence"[Title/Abstract]
 OR "large language model*"[Title/Abstract] OR LLM[Title/Abstract] OR LLMs[Title/Abstract]
 OR ChatGPT[Title/Abstract] OR chatbot*[Title/Abstract] OR "AI chatbot*"[Title/Abstract])
)
AND
(
 "Health Information Seeking Behavior"[Mesh]
 OR health[Title/Abstract] OR medical[Title/Abstract] OR patient*[Title/Abstract]
 OR symptom*[Title/Abstract] OR treatment*[Title/Abstract] OR disease*[Title/Abstract]
 OR medication*[Title/Abstract] OR diagnosis[Title/Abstract] OR "mental health"[Title/Abstract]
 OR "healthcare"[Title/Abstract] OR "health care"[Title/Abstract]
)
AND
(
 "information seeking"[Title/Abstract] OR "health information seeking"[Title/Abstract]
 OR "online health information"[Title/Abstract] OR trust[Title/Abstract]
 OR credibility[Title/Abstract] OR reliance[Title/Abstract] OR verification[Title/Abstract]
 OR adoption[Title/Abstract] OR "intention to use"[Title/Abstract]
 OR "continued use"[Title/Abstract] OR "source switching"[Title/Abstract]
 OR "help seeking"[Title/Abstract]
)
AND ("2022/01/01"[Date - Publication] : "3000"[Date - Publication])
AND English[lang]

**Scopus**

Search date: January 8, 2026
Platform: Scopus/Elsevier
Limits: Publication year > 2021
Search strategy:

TITLE-ABS-KEY(

(

"generative AI"

OR "generative artificial intelligence"

OR "large language model"

OR "large language models"

OR ChatGPT

OR chatbot*

)

AND

(

health

OR medical

OR patient*

OR "mental health"

)

AND

(

"information seeking"

OR trust

OR verification

OR adoption

OR reliance

)

)

AND PUBYEAR > 2021

**PsycINFO**

Search date: January 8, 2026
Platform: PsycINFO via EBSCOhost
Limits: English; publication date from 2022 onward
Search strategy:

AB((ChatGPT OR chatbot* OR "large language model" OR "large language models" OR "generative AI") AND (health OR medical OR patient* OR "mental health") AND ("information seeking" OR trust OR adoption))

OR

TI((ChatGPT OR chatbot* OR "large language model" OR "large language models" OR "generative AI") AND (health OR medical OR patient* OR "mental health") AND ("information seeking" OR trust OR adoption))

**Web of Science Core Collection**

Search date: January 8, 2026

Platform: Web of Science/Clarivate

Limits: English; publication years 2022–2026

Search strategy:

TS=(("generative AI" OR "generative artificial intelligence" OR ChatGPT OR chatbot* OR "large language model" OR "large language models" OR LLM OR LLMs) AND (health OR medical OR patient* OR symptom* OR treatment* OR diagnosis OR "mental health") AND ("information seeking" OR "health information seeking" OR trust OR verification OR reliance OR adoption OR "intention to use"))

**IEEE Xplore**

Search date: January 8, 2026
Platform: IEEE Xplore
Limits: 2022-present; English
Search strategy:

("All Metadata":"ChatGPT" OR "All Metadata":"generative AI" OR "All Metadata":"large language model" OR "All Metadata":"large language models" OR "All Metadata":"chatbot") AND ("All Metadata":"health" OR "All Metadata":"medical" OR "All Metadata":"patient" OR "All Metadata":"mental health") AND ("All Metadata":"information seeking" OR "All Metadata":"trust" OR "All Metadata":"verification" OR "All Metadata":"adoption")

**ACM Digital Library**

Search date: January 8, 2026
Platform: ACM Digital Library
Limits: Publication date 2022-present; English
Search strategy:

("generative AI" OR "generative artificial intelligence" OR ChatGPT OR chatbot* OR "large language model" OR "large language models" OR LLM OR LLMs)

AND

(health OR medical OR patient* OR symptom* OR treatment* OR diagnosis OR "mental health")

AND

("information seeking" OR "health information seeking" OR trust OR verification OR reliance OR adoption OR "intention to use")

**ERIC**

Search date: January 8, 2026
Platform: ERIC via EBSCOhost
Limits: 2022-present; English; peer reviewed
Search strategy:

(ChatGPT OR "generative AI" OR "large language model" OR "large language models" OR chatbot*)

AND

(health OR medical OR patient* OR "mental health")

AND

("information seeking" OR trust OR verification OR adoption OR reliance)

**EBSCO**

Search date: January 8, 2026
Platform: EBSCOhost
Limits: English; published date 2022-2026
Search strategy:

TX ("generative AI" OR "generative artificial intelligence" OR "large language model*" OR LLM OR LLMs OR ChatGPT OR chatbot* OR "AI chatbot*")
AND
TX (health OR medical OR patient* OR symptom* OR treatment* OR disease* OR medication* OR diagnosis OR "mental health" OR healthcare OR "health care")
AND
TX ("information seeking" OR "health information seeking" OR "online health information" OR trust OR credibility OR reliance OR verification OR adoption OR "intention to use" OR "continued use" OR "source switching")

**ProQuest**

Search date: January 8, 2026
Platform: ProQuest
Limits: English; publication date from 2022 onward
Search strategy:

ab,ti(("generative AI" OR "generative artificial intelligence" OR "large language model*" OR LLM OR LLMs OR ChatGPT OR chatbot* OR "AI chatbot*"))
AND
ab,ti((health OR medical OR patient* OR symptom* OR treatment* OR disease* OR medication* OR diagnosis OR "mental health" OR healthcare OR "health care"))
AND
ab,ti(("information seeking" OR "health information seeking" OR "online health information" OR trust OR credibility OR reliance OR verification OR adoption OR "intention to use" OR "continued use" OR "source switching"))

**Google Scholar**

Search date: January 8, 2026
Platform: Google Scholar
Limits: Publication date from 2022 onward; English-language records screened
Search strategy:

Google Scholar was searched using simplified phrase-based queries because long Boolean strings are handled inconsistently. The following phrases were searched manually: “generative AI health information seeking”, “ChatGPT health information seeking”, “large language model health information seeking”, “AI chatbot health information seeking”, “generative AI patient health information”, “ChatGPT patient information seeking”, “large language model patient information seeking”, and “AI chatbot health advice”. Results were screened manually according to the predefined eligibility criteria.
