# Supplementary material for: Consumer and Patient Health Information Seeking With Generative AI Tools: Scoping Review of Facilitators and Barriers
Source: J Med Internet Res. 2026 Sep 2;28:e93944. doi: 10.2196/93944 (PMC13583531; doi:10.2196/93944)
Supplement: Supplementary file 3 [file jmir_v28i1e93944_app3.docx]

*Multimedia Appendix 3: Characteristics and Charted Findings of Included Studies*

|  | Author(s) | Title | Population | Domain | Study design | AI tools | Sample | Findings | Factors identified |
| --- | --- | --- | --- | --- | --- | --- | --- | --- | --- |
| 1. | Adeyemi et al., 2025 [36] | Patterns of AI Used for Generating Health Information among Students in South West Nigeria | University students | Preventive, self-care | Survey based study | ChatGPT, Meta AI, Gemini, DeepSeek, Copilot, Claude, Bing AI | N = 204 | - High awareness of AI tools (99.0%), most students reported using AI to generate health information (89.2%). - Topics searched/generated with AI: drug information, diet/nutrition, exercise/fitness, and mental health. | **Facilitators**   - Students viewed AI-generated health information as moderately reliable. - Clinicians as trusted reference point.   **Barriers / limiting factors**   - Students trusted clinicians more than AI. - Demographics did not explain differences. |
| 2. | Alanez, 2025  [37] | Examining the role of ChatGPT in  promoting health behaviors and lifestyle  changes among cancer patients | Cancer patients | Self-management, behaviour change | Qualitative focus group | ChatGPT | N = 72 | - ChatGPT helped to better understand cancer-related information. - Improved health literacy: clearer/ accessible language. - ChatGPT as useful for locating or being directed to additional resources and support options. ChatGPT helped worries, correct misconceptions around treatments and lifestyle/prevention topics. | **Facilitators**   - Clarity and accessibility of explanations. - Perceived usefulness for self-management and lifestyle change. - Emotional/informational support - Ability to point users to external resources, supporting social/practical support-seeking.   **Barriers / limiting factors**   - Privacy concerns; limited personalisation; generic responses; Reliability/accuracy. |
| 3. | Al-Anezi, 2023 [38] | Exploring the use of ChatGPT as a virtual health coach for chronic disease management | Chronic disease patients | self-managemen, health, behaviour change | Qualitative quasi-experimental study | ChatGPT (free version; GPT-3.5) | N = 29 | - ChatGPT as useful for chronic disease self-management and health education, mainly as supplement. - Perceived benefits alongside significant concerns about safety, accuracy, and appropriateness for medical decision-making. | **Facilitators**   - Improved health literacy, accessibility, convenience; perceived cost-effectiveness. - Support for behaviour change - Scalability for wider reach.   **Barriers**   - No physical examination/clinical assessment. - Lack of human connection. - Accuracy/reliability/ethical concerns |
| 4. | Al Shboul et al., 2024 [39] | Investigating the Use of ChatGPT as a Novel Method for Seeking Health Information: A Qualitative Approach | General population | User experience | Qualitative study | ChatGPT | N = 16 | - Participants described using ChatGPT as a fast, convenient, easy-to-use way to get answers, especially for basic or general health questions and quick clarification. - They were cautious about relying on it for serious, complex, or urgent medical issues, emphasizing that it cannot replace clinical assessment or personalised professional advice. - Credibility, misunderstandings, privacy/security worries, impersonal. | **Facilitators**   - Convenience and accessibility. - Ease of use and natural-language interaction. - Perceived usefulness for basic questions, initial orientation, and general explanations.   **Barriers**   - Trust/credibility and accuracy concerns. - Not suitabe for complex/high-stakes situations. - Lack of personalisation and limited empathy/emotional support. - Risk of misinterpretation/ miscommunication. - Privacy and security concerns. |
| 5. | Altamimi1et al., 2024 [11] | Exploring online health resources and self-care among irritable bowel syndrome patients: Analyzing internet use and AI chatbot interactions | Irritable bowel syndrome patients | Health literacy/ preference | Survey based study | AI chatbots | N = 451 | - Use of AI chatbots for health information was relatively low compared to search engines and official health websites. - Participants rated AI chatbot information as moderate in quality, lower than official health sources but higher than social media. - Use of AI chatbots was associated with higher overall rates of health information searching. | **Facilitators**   - Accessibility/convenience via mobile devices. - Authoritative/trusted sources,   **Barriers**   - Trust concerns regarding information quality. - Relatively low adoption of AI chatbots compared to traditional digital sources. - Digital divide factors (age, income, employment status) shaping frequency and intensity of information seeking. |
| 6. | Ayo-Ajibola et al., 2024 [21] | Characterizing the adoption and experiences of users of Artificial Intelligence-generated health information in the United States: Cross-sectional questionnaire study | General population | Health literacy/ preference | Survey based study | ChatGPT | N = 2406 | - 21.5% used ChatGPT; 39.3% used it 2–3 per week. - Main uses: decide if consultation is needed (47.4%); explore alternative treatments (46.2%). - 67.9% suspected inaccuracy; 67.5% brought info to a physician. - Actions: requested referral/test (35.6%); medications (31.0%). | **Facilitators**   - Younger age, lower education ,lower income. - Heavier general online health info seeking. - **Barriers** - Accuracy concerns: 67.9% suspected inaccuracy. - Lower eHealth literacy among users. - Reliance on urgent care contexts. |
| 7. | Bosco et al., 2024 [12] | Testing 3 modalities (voice assistant, chatbot, and mobile App) to assist older African American and black adults in seeking information on Alzheimer disease and related dementias: Wizard of Oz usability study | Blach older adults with Alzheimer | Health literacy/ preference | Qualitative usability study | Voice assistant, chatbot, and mobile app prototypes | N = 55 | - Preferred information written in simple language, delivered quickly. - Wanted features that let them save, revisit, and share information easily. - Trust depended on clearly showing a credible health sources. - Engagement increased when content felt culturally relevant and reflected older Black/African American adults’ experiences and concerns. | **Facilitators**   - Design: simple language; credible sourcing; culturally tailored content; save/store/share features; time efficiency; interaction design that increases control/representation.   **Barriers**   - Design: complex/convoluted language; unclear credibility; lack of cultural relevance; inability to save/share; inefficient interaction; interaction that reduces control/representation. |
| 8. | Branley-Bell et al., 2023 [22] | Chatbots for embarrassing and stigmatizing conditions: Could chatbots encourage users to seek medical advice? | General adult population | Health literacy/ preference | Survey based study | Chatbots | N = 402 | - Participants preferred consulting a doctor rather than a chatbot for an initial health consultation. - Preference for chatbot use higher for embarrassing sexual symptoms. - Participants who preferred chatbots believed they would seek medical advice earlier than those who preferred doctors. - Preferred in-person for doctors, and text-based interaction for chatbots. | **Facilitators**   - Convenience and ease: chatbots as quicker and easier to access than arranging a doctor visit. - Reduced social discomfort: chatbots as less embarrassing/stressful than talking a doctor.   **Barriers**   - Trust and quality concerns: doctors perceived as more accurate, trustworthy, reassuring, useful, and confidential than chatbots. - Interaction friction: chatbots were perceived as more frustrating to use. |
| 9. | Brewer, 2024 [13] | Understanding voice-based information uncertainty: A case  study of health information seeking with voice assistants | General adult population | Health literacy/ preference | Qualitative study | Voice assistants | N =30 | - Voice-based health searching often produces high uncertainty because users cannot easily assess credibility without visual cues. - Users rely on voice assistants mainly for simple, low-stakes questions and shift to other sources/devices for complex or risky issues. - When uncertain, users commonly rephrase queries, verify elsewhere, or switch to screen-based search; many want sources or a transcript to support verification. | **Facilitators**   - Convenience and speed of voice interaction. - Source transparency increases confidence. - Features that support verification reduce uncertainty.   **Barriers**   - Lack of visual credibility cues and limited context reduces trust calibration. - One-shot spoken answers increase cognitive load (hard to skim, compare, or revisit). - Misinterpretation risk and unclear sourcing heighten uncertainty, especially for high-stakes health topics. |
| 10. | Chen et al., 2025 [27] | Utilisation of AI-driven chatbots for perioperative health information seeking: A descriptive qualitative study of orthopaedic patients and family members | Orthopaedic surgery patients and family members | Self-care advice and decision support | Qualitative study | AI-driven chatbots | N = 13 | - Participants preferred AI chatbots over search engines for questions; chatbots gave direct answers, no through links/ads. - Chatbots perceived as improving access and quality of perioperative information (fast, available anytime, detailed guidance). - Participants preferred asking chatbots over asking clinicians; interaction felt non-judgmental and emotionally safe. - Response quality depended on how well they prompted; more specific/context-rich questions produced better answers. | **Facilitators**   - Efficiency: faster, more targeted retrieval than search engines. - Personalisation: advice tailored to surgery type, recovery stage, context. - Practical clarity: mediated-actionable guidance. - Accessibility: 24/7 availability; reduces waiting/clinic barriers. - Interaction comfort: non-judgmental, patient listener, reduces anxiety and hesitation. - Iterative dialogue: ability to ask follow-ups and refine questions to get more useful guidance.   **Barriers**   - Accuracy/specificity risks: answers become generic/less reliable when prompts are vague. - Prompting skills. - Chatbot should be reviewed/ updated and users may need structured tutorials. |
| 11. | Esmaeilzadeh et al., 2025 [24] | Using AI chatbots (e.g., CHATGPT) in seeking health-related information online: The case of a common ailment | Low back pain patients | Self-care advice and decision support | Survey based study | ChatGPT | N = 231 | - Both utilitarian value and hedonic value significantly increased satisfaction with ChatGPT for health information. - Satisfaction strongly predicted continued intention to use and willingness to act on ChatGPT’s health advice - Satisfaction mediated the relationship between perceived value and behavioral intentions. - Younger age, higher education, and greater AI experience increased likelihood of reuse and action. | **Facilitators**   - Ease of use and conversational interaction. - Perceived accuracy, relevance, clarity, and completeness of information. - Time saving and cost-effective access to health information; Privacy - sensitive health questions. - Emotional reassurance and engagement during interaction.   **Barriers**   - Concerns: accuracy/reliability for decision-making; Lack of clinical validation and professional oversight. - Limited suitability for emergency or high-risk conditions; Dependence on user ability to ask effective question. |
| 12. | Guo et al., 2025 [9] | Promoting trust and intention to adopt health information generated by  ChatGPT among healthcare customers: An empirical study | Healthcare customers | Health literacy/ preference | Survey based study | ChatGPT | N = 308 | - Trust strongly predicted intention to adopt ChatGPT-generated health information. - Trust increased with situational normality, information quality, autonomy, and personalisation. - Social influence was negatively associated with trust. - Familiarity with ChatGPT was a significant control. | **Facilitators**   - Situational normality: feels expected or appropriate to use ChatGPT. - Information quality and control/ independence in seeking information. - Personalisation and familiarity with ChatGPT   **Barriers.**   - When use is driven by others’ opinions, trust in ChatGPT decreases. |
| 13. | Harrington & Egede, 2023 [25] | Trust, comfort and relatability: Understanding black older adults’ perceptions of chatbot design for health information seeking | Black older adults | Health literacy/ preference | Qualitative study | Health information chatbot / embodied conversational agent | N = 30 | - Participants viewed health chatbots as useful and learnable for finding health information quickly. - Trust shaped by professional credibility cues (e.g., doctor title/ professional appearance). - Racial likeness (a Black chatbot) increased comfort/relatability, but not automatically increase trust and sometimes raised skepticism if the source behind chatbot felt unclear. | **Facilitators**   - Perceived usefulness and ease of learning/using a chatbot. - Professional identity cues that signal credibility and increase trust. - Representation/likeness that increases comfort and willingness to engage.   **Barriers**   - Low tech confidence and limited prior chatbot experience. - Skepticism when identity cues feel cosmetic or when it’s unclear who provides/validates the information. - Baseline mistrust in healthcare systems that is not resolved by representation alone. |
| 14. | Lau et al., 2025 [40] | Exploring the acceptance and opportunities of using a specific Generative AI chatbot to assist parents in managing pediatric rheumatological chronic health conditions: Mixed methods study | Children with chronic pediatric rheumatologic conditions and parents | Chronic illness managemen | Mixed methods | ChatGPT | N = 47 | - Parents and children reported higher intention to use a condition-specific chatbot than ChatGPT for managing the child’s condition. - Awareness and use were mixed: 70% of parents heard of ChatGPT, 39% used AI; all children/young people heard of it and 45% used it. - Confidence in using GenAI was low. | **Facilitators**   - Accessibility: quick support between appointments when professionals are not available. - Specificity and fit: Higher intentions when chatbot tailored to pediatric rheumatology needs and anchored in clinician-endorsed content.   **Barriers**   - Trust and credibility concerns about ChatGPT for health: Participants worried about unverified sources and inaccurate or unreliable output for complex medical information. - Low confidence using GenAI. - Design mismatch risk: Parents and children/young people were seen as distinct user groups with different needs, so a one-size design could reduce acceptance. |
| 15. | Link & Beckmann, 2025 [20] | AI at everyone’s fingertips? Identifying the predictors of health information seeking intentions using AI | General adult population | Intentions to use | Survey based study | AI for health information seeking, framed as AI chatbots | N = 1121 | - AI-related subjective norms, and perceived seeking control significant positive predictors of intention to seek health information via AI. - Trust in AI was a statistically significant but weak positive predictor of intention. - Affective AI-risk perceptions (feeling worried/scared/uncertain) reduced intention, while cognitive | **Facilitators**   - Positive attitudes: seeing AI HIS as beneficial/helpful. - Higher perceived seeking control. - Perceiving AI health seeking as acceptable/ common among peers. - Higher trust in AI.   **Barriers**   - Affective responses: worry, fear, uncertainty. - Higher cognitive risk perceptions. |
| 16. | McLeod et al., 2025 [28] | Opening the digital doorway to sexual  healthcare: Recommendations from a behaviour change wheel analysis of barriers  and facilitators to seeking online sexual health  information and support among underserved  populations | Under-served adult population | Intentions to use, behavior change | Qualitative study | AI for health information seeking, framed as AI chatbots or automated responses | N = 35 | - Participants often experienced chatbots/automated responses as a barrier when they were used instead of a human, especially for sensitive, complex, or emotionally loaded sexual health needs. - Chatbots/automation as impersonal and frustrating. - Automation interacted with other barriers, reduced willingness to use digital channels. | **Facilitators**   - Fast, responsive support and low effort access; automation acceptable if improves speed and does not replace human contact when needed. - A clear pathway if escalates to a human.   **Barriers**   - No human connection / lack of empathy. - Fear of miscommunication or not being understood by an automated system. - Low confidence that responses are appropriate. - Frustration when automation blocks access to a person or prolongs resolution. |
| 17. | Mendel et al., 2025 [5] | Laypeople’s use of and attitudes toward large language models and search engines for health queries: Survey study | General adult population | Intentions to use | Survey based study | Primarily ChatGPT; some use of other tools e.g., Bard/ Copilot. | N = 2002 | - Search engines more common for health searches (95.6% vs 32.6%). - Search engines as the first source consulted (62%), LLMs (14%). - More LLM: men, Black, Asian, good health, higher tech proficiency. - Similar likelihood of using LLMs vs search engines for symptoms, treatment, preventive care, diagnoses, test-result interpretation. - Search engines for administration. - LLMs perceived less useful/relevant/ biased but elicited fewer negative feelings, perceived more human-like. | **Facilitators**   - Lower perceived bias and advertising influence. - Fewer negative feelings during use. - Human-like interaction (perceived anthropomorphism). - Ease of use comparable to search engines.   **Barriers**   - Lower perceived usefulness and relevance for answering health questions. - LLMs viewed as less able than search engines to replace a doctor for answering health questions. |
| 18. | Miqdadi et al., 2025 [29] | Attitudes toward seeking professional psychological help, self-stigma of seeking help, and preferences for ai mental health chatbots vs. human counsellors: A mixed methods study. | Nursing students | Help seeking | Mixed methods | AI mental health chatbots | N = 757 | - Sudents preferred human counsellors (61%), less preferred AI mental health chatbots (32%). - Students favored chatbots: emphasize anonymity/accessibility as advantages for help/information. - Students favored human counsellors highlighted trust, emotional support, and cultural sensitivity. | **Facilitators**   - Accessibility to support/information. - Anonymity: reduced exposure/discomfort when seeking sensitive mental health information.   **Barriers**   - Preference for human support: stronger trust, emotional support, and cultural sensitivity. - Stigma, low perceived need, limited awareness of services, and mistrust in care quality. |
| 19. | Qiu et al., 2026 [2] | Understanding users’ online health information seeking switching intention: From traditional health information platforms to GAI | General adult population | Intention to use | Mixed methods | Generative AI | N = 834 | - Push factors: health anxiety, usage fatigue, privacy concerns. - Significantly increased intention to switch to GenAI. - Pull factors: perceived convenience, perceived mind, information quality. - Social influence: increased switching intention, moderated health anxiety, usage fatigue, convenience, information quality. | **Facilitators (switching intention to GAI):**   - Push: health anxiety; usage fatigue; privacy concerns. - Pull: perceived convenience; perceived “mind”; information quality. - Mooring: social influence. |
| 20. | Reinhardt et al., 2025 [30] | Help me, Doctor AI? A cross-national experiment on the effects of disease  threat and stigma on AI health information-seeking intentions | General adult population | Intention to use | Quantitative experiment | Chatbots | N = 1951 | - Disease threat/stigma did not increase intention to use GenAI. - Country differences. - AI trust and literacy: strongest predictors of GenAI intention. - People preferred doctors over GenAI; for mild/stigmatized conditions, GenAI was preferred. | **Facilitators**   - Higher AI trust and literacy. - Younger age, country context. - Mild/stigmatized condition increased preference for GenAI over friends.   **Barriers**   - Overall preference for doctors over GenAI. |
| 21. | Rojkovich et al., 2024 [26] | The use of ChatGPT for online health information seeking in Hungary | General adult population | Intention to use | Survey based study | ChatGPT | N = 313 | - Significant predictors: information-seeking skills, expertise, transparency, subjective norm, perceived risk/reliability/ understandability. - Positive effects: expertise, transparency, subjective norm, perceived reliability, perceived understandability. - Negative effects: perceived risk, information-seeking skills. | **Facilitators (higher intention to use ChatGPT for health info)**   - Subjective norm (social pressure/acceptance) - Perceived reliability. - Perceived understandability (feeling one understands how it works). - Expertise (belief it provides professional answers). - Transparency (smaller positive role).   **Barriers / inhibitors**   - Perceived risk (higher risk- lower intention). - Information-seeking skills (higher skill- slightly lower intention; interpreted as skilled seekers being more cautious/less likely to rely on ChatGPT). |
| 22. | Shi et al., 2025 [1] | Empowering individuals to adopt artificial intelligence for health  information seeking: A latent profile analysis among users in Hong Kong | General adult population | Intention to use | Survey based study | AI | N = 1051 | Five user profiles: (1) Discreet approchers: Positive attitudes, low norms and self-efficacy; (2) Casual investigators: favorable attitudes/ strongest norms; lowest tech anxiety; high trust/accuracy; high intention; (3) Apprehensive moderates: cautious toward AI; (4) Apathetic bystanders: Lowest motivation/ norms; (5) Anxious explorers: High intention, trust/accuracy, self-efficacy, high technology anxiety. | **Facilitators**   - Positive attitudes toward AI HIS. - Stronger norms, higher self-efficacy/past use. - Higher trust and perceived accuracy. - Demographic: Younger age, higher income.   **Barriers**   - Higher technology anxiety. - Low descriptive norms/self-efficacy, cautious. - Demographic: Women -cautious, older age.Top of Form   Bottom of Form |
| 23. | Sun et al., 2025 [6] | Interface Matters: Exploring human trust in health information from large language models via text,  speech, and embodiment | General adult population | Intentions to use | Mixed methods | GPT-4 for text chat, speech and embodied (all three) | N = 20 | - Trust in information by interface: text more trusted than embodied. - Trust in information linked to trust in the interface and overall usability. - Intention to use the system highest for text, lower for speech, lowest for embodied. | **Facilitators**   - High usability and sense of control. - Interface trust: predictable/dependable - Text support verification: reread/compare/share - Embodiment increase engagement/follow-up.   **Barriers**   - Harder verification in speech/embodied modes. - Missing or unreliable sourcing/citations. - Privacy concerns with present agents. - Generic answers; lack of personalization. |
| 24. | Svestkova et al., 2025 [23] | Factors that influence trust and willingness to use generative AI for health information: A cross-sectional study | General adult population | Intentions to use | Survey based study | Generative AI | N = 4775 | - Trust in AI health information:strong predictor of willingness to use AI. - Health status/anxiety related to willingness through trust. - eHealth literacy marginally related to trust, not related to willingness. - People use AI for similar health questions they search online. | **Facilitators**   - Perceived credibility and reliability of the AI. - Prior GenAI familiarity/experience. - Existing online health-search habits. - Health motivation- confidence in AI channel.   **Barriers**   - Doubts about credibility make AI a last resort or something users avoid for health decisions. - eHealth literacy does not improve comfortable using AI. |
| 25. | Wardle et al., 2025 [7] | Evolving health information–seeking behavior in the context of Google AI overviews, ChatGPT, and Alexa: Interview study using the think-aloud protocol | General adult population | Intentions to use | Qualitative think-aloud study | Google search with AI Overviews, ChatGPT (mainly 3.5), and Amazon Alexa (device or app). | N = 27 | - Participants used AI as mix-and-match search routine. - ChatGPT used for quick orientation (summaries, keyword generation) even when unsure about accuracy. - Google AI Overviews triggered caution; users skipped and relied on conventional results. - Alexa as useful for quick, low-stakes queries, not for deeper questions. | **Facilitators**   - Clear summaries help users get oriented. - Conversational follow-up to refine questions. - Keyword generation for subsequent searching. - Convenience of voice for simple tasks.   **Barriers**   - Lack of visible sources/citations; concerns about bias or inaccuracies. - Voice-channel limitations for complex topics. - Usability breakdowns with voice assistants. - Multilingual and cultural-fit issues. |
| 26. | Yin & Neyens, 2024 [41] | Examining how information presentation methods and a chatbot impact the use and effectiveness of electronic health record patient portals: An exploratory study | University students | Intentions to use | Experimental qauntitative study | Chatbot with inflammatory bowel disease (IBD) content | N = 92 | - Video increased search time, improved perceived informedness. - Text faster for locating; shortest in text/chatbot condition. - Prior chatbot experience/knowing someone with IBD associated with faster searching. - 50% in chatbot conditions used it, when struggled to find answer. | **Facilitators**   - Video presentation more informed/engaged. - Text presentation faster/efficient searching. - Chatbot experience, topic familiarity, HIS motivation.   **Barriers**   - Higher cognitive load formats slow search. - Low chatbot uptake unless users get stuck. |
| 27. | Zeng et al., 2025 [42] | Perceptions and attitudes of Chinese oncologists toward endorsing AI-driven chatbots for health information seeking among patients with cancer: Phenomenological qualitative study | Oncologists | Perceived usefulness | Qualitative study | AI chatbots | N = 24 | - Benefits: accessibility/convenience; support chronic illness management. - Concerns: liability/accountability, misinformation (including outdated/incorrect answers), lack of personalization, privacy/data security, patient readiness/. - Chatbots improve consultation efficiency, but overreliance can erode trust and create tension. | **Facilitators**   - Access for non-urgent questions; workload reduction; Support for illness care/management.   **Barriers / concerns**   - Unclear liability/accountability if harm occurs. - Misinformation/hallucinations; over simplification; higher risk for low literacy. - Limited personalization, lack of patient context. - Privacy/data security concerns. - Patient readiness (age/digital literacy). |
